# A Preliminary Investigation of Similarities of High Resolution Cervical Auscultation Signals Between Thin Liquid Barium and Water Swallows

**DOI:** 10.1109/JTEHM.2021.3134926

**Published:** 2021-12-10

**Authors:** Ryan Schwartz, Yassin Khalifa, Erin Lucatorto, Subashan Perera, James Coyle, Ervin Sejdić

**Affiliations:** Department of Electrical and Computer EngineeringSwanson School of EngineeringUniversity of Pittsburgh 6614 Pittsburgh PA 15261 USA; Department of Communication Science and DisordersSchool of Health and Rehabilitation SciencesUniversity of Pittsburgh 6614 Pittsburgh PA 15260 USA; Division of Geriatric MedicineDepartment of MedicineUniversity of Pittsburgh 6614 Pittsburgh PA 15261 USA; The Edward S. Rogers Department of Electrical and Computer EngineeringFaculty of Applied Science and EngineeringUniversity of Toronto 7938 Toronto ON M5S 2E4 Canada; North York General Hospital 8613 Toronto ON M2K 1E1 Canada

**Keywords:** Cervical auscultation, dysphagia, hyoid displacement, signal processing, swallowing

## Abstract

Dysphagia, commonly referred to as abnormal swallowing, affects millions of people annually. If not diagnosed expeditiously, dysphagia can lead to more severe complications, such as pneumonia, nutritional deficiency, and dehydration. Bedside screening is the first step of dysphagia characterization and is usually based on pass/fail tests in which a nurse observes the patient performing water swallows to look for dysphagia overt signs such as coughing. Though quick and convenient, bedside screening only provides low-level judgment of impairment, lacks standardization, and suffers from subjectivity. Recently, high resolution cervical auscultation (HRCA) has been investigated as a less expensive and non-invasive method to diagnose dysphagia. It has shown strong preliminary evidence of its effectiveness in penetration-aspiration detection as well as multiple swallow kinematics. HRCA signals have traditionally been collected and investigated in conjunction with videofluoroscopy exams which are performed using barium boluses including thin liquid. An HRCA-based bedside screening is highly desirable to expedite the initial dysphagia diagnosis and overcome all the drawbacks of the current pass/fail screening tests. However, all research conducted for using HRCA in dysphagia is based on thin liquid barium boluses and thus not guaranteed to provide valid results for water boluses used in bedside screening. If HRCA signals show no significant differences between water and thin liquid barium boluses, then the same algorithms developed on thin liquid barium boluses used in diagnostic imaging studies, it can be then directly used with water boluses. This study investigates the similarities and differences between HRCA signals from thin liquid barium swallows compared to those signals from water swallows. Multiple features from the time, frequency, time-frequency, and information-theoretic domain were extracted from each type of swallow and a group of linear mixed models was tested to determine the significance of differences. Machine learning classifiers were fit to the data as well to determine if the swallowed material (thin liquid barium or water) can be correctly predicted from an unlabeled set of HRCA signals. The results demonstrated that there is no systematic difference between the HRCA signals of thin liquid barium swallows and water swallows. While no systematic difference was discovered, the evidence of complete conformity between HRCA signals of both materials was inconclusive. These results must be validated further to confirm conformity between the HRCA signals of thin liquid barium swallows and water swallows.

## Graphical abstract

High resolution cervical auscultation (HRCA) is a sensor-based method that is being investigated as a non-invasive method to diagnose swallowing disorders. HRCA signals are usually collected simultaneously with videofluoroscopy exams which are performed using barium boluses including thin liquid to acquire a visual proof of swallowing abnormalities. All research conducted for using HRCA in dysphagia is based on thin liquid barium boluses and thus not guaranteed to provide valid results if used for water boluses commonly used in bedside screening. If HRCA signals show no significant differences between water and thin liquid barium boluses, then the same algorithms developed on thin liquid barium boluses used in diagnostic imaging studies, it can be then directly used with water boluses. This study investigates the similarities and differences between HRCA signals from thin liquid barium swallows compared to those signals from water swallows.

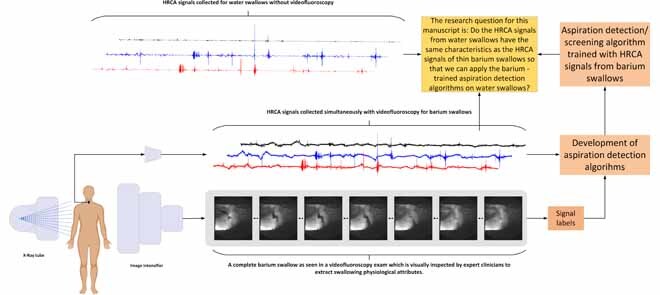

## I. Introduction

Swallowing is the systematic, complex series of events during which food and liquid are transferred from the mouth to the stomach [1]. Oropharyngeal swallowing requires precise biomechanical and neurological coordination of over 30 pairs of muscles, numerous peripheral nerves, and displacement of structures to guarantee adequate and safe passage of materials through the upper aerodigestive tract [2]. Oropharyngeal dysphagia (OPD), also known as difficulty swallowing, frequently occurs in the setting of a variety of illnesses, injuries, and disorders that disrupt this coordination. These include neurological conditions (e.g., stroke, Parkinson’s disease, multiple sclerosis, ALS), injuries (e.g., traumatic brain injury, maxillofacial fractures), head and neck cancer, chronic or degenerative illness (e.g., scleroderma, systemic organ disease) iatrogenic etiologies (e.g., cardiothoracic procedures), and others [2]–[4]. Patients with OPD are at elevated risk for aspiration. Aspiration is the entry of gravity-dependent foreign material through the larynx and into the trachea (i.e., below the true vocal folds). Patients with OPD who aspirate are seven times more likely to develop pneumonia versus individuals who do not [5]. More than half of individuals who reside in an institution, such as an assisted living facility or skilled nursing facility, experience OPD [6], [7]. With elderly patients, dysphagia often contributes to other adverse and complicating conditions, including weight loss, nutritional deficiency, dehydration and others [6], [8].

Bedside observation (screening) of the patient’s swallowing is frequently the first step in comprehensive swallowing assessment. This is typically performed by nursing or other medical staff. OPD screening involves non-instrumental pass-fail procedures that are completed via the administration of water boluses. During these trials, the screener notes the presence or absence of overt signs of dysphagia, such as coughing or choking [9]. Examples of formalized bedside swallowing screening protocols include the Toronto Bedside Swallow Screening Test [10], the Yale Swallow Protocol [11], and the modified Mann Assessment of Swallowing Ability [12]. In screens solely dependent on the presence of cough after swallowing, reduced sensitivity plagues the process, particularly in the case of “silent” aspiration which occurs in up to 89% of people who exhibit aspiration during imaging tests [13], [14]. Silent aspiration occurs without overt clinical indicators of aspiration (e.g., coughing, choking, “wet” vocal quality) [4]. In the case of screen failure and/or inconclusive results (i.e., an absence of overt signs of aspiration, though clinical concern remains for silent aspiration), the patients are generally referred to a speech-language pathologist (SLP) for a swallowing assessment. Additional assessment via diagnostic imaging tests such as videofluoroscopic swallowing study (VFSS) and fiberoptic endoscopic evaluation of swallowing may also be required [15].

VFSS is also known as a modified barium swallow study and is completed by administering trials of a barium sulfate suspension of various consistencies. During these trials, a series of real-time radiographic video images of the oral cavity and the upper aerodigestive tract is captured during the swallow. VFSS is considered the gold standard for dysphagia diagnosis and is the most available imaging study for OPD; however, for certain patients it may be delayed due to accessibility, undesirable, unfeasible, or completely unavailable, leaving them undiagnosed or incorrectly diagnosed. This diagnostic barrier leaves the patient vulnerable to dysphagia-related complications [16], [17]. Therefore, there is a high demand for a widely accessible dysphagia assessment utility that can perform accurate screening and provide insight regarding underlying swallowing physiology [18]–[21].

High resolution cervical auscultation is an emerging method that has recently been utilized as a less expensive and non-invasive swallowing screening and assessment tool compared with traditional diagnostic imaging tests. HRCA involves the use of neck sensors (i.e., a 3-dimensional accelerometer to record vibrations and a contact microphone) to record sound induced by the swallowing process. Raw HRCA signals are subject to movement, coughing, speaking, or external vibrations [22], [23]. Unlike VFSS, the noisy nature of HRCA signals makes their visual interpretation by clinicians extremely difficult due the presence of other signal components originating from different physiological processes such as coughing and head movement. On the other hand, advanced signal processing and machine learning techniques have produced several sets of preliminary evidence confirming the precision of automatic interpretation of HRCA signals in the detection of swallowing kinematic events and airway protection during swallowing [15], [24]–[28]. For instance, HRCA has been shown to accurately track the hyoid bone throughout the duration of a swallow without assistance or supervision from human experts, with similar accuracy to these experts [27], [29]. Further, this technology has demonstrated the ability to reliably detect upper esophageal (UES) opening and closure, and laryngeal vestibule (LV) closure and reopening [16], [17], [28], [30].

The levels of accuracy and reliability in measurement and detection that HRCA has attained have been confirmed via machine learning based techniques utilizing VFSS tests which require the use of a contrast agent (i.e., barium sulfate suspension). Conversely, bedside swallow screening protocols are completed with water and using a barium suspension for this purpose is clinically unrealistic. In order to establish HRCA’s reliability as a screening tool in the clinical context, it must demonstrate the same level of accuracy and insight for water as it does for barium.

The machine learning algorithms trained using HRCA signals to perform the kinematic analysis in swallowing are called supervised-learning algorithms. This means the algorithms need reference data to be trained. The references must be created using barium swallows, because simultaneous VFSS recordings are needed to complete analysis of kinematics and penetration/aspiration. One way to achieve HRCA water-based bedside screening is to train machine learning algorithms using HRCA signals from the correctly rated barium swallows and test those algorithms on HRCA signals from water-based swallows. However, HRCA bedside screening performed using water can only be used as a reasonable adjunct if there are no significant HRCA signal differences between water and barium swallows.

An HRCA bedside screening performed using water has the potential for numerous advantages over other screening protocols and/or subsequent diagnostic testing. HRCA screening will not incur a significant training burden on caregivers, which may range from nurses to SLPs. Specific education may be conducted during usual departmental trainings, and will include sensor placement and familiarity with the analysis app. Given these potential advantages, this study investigates whether HRCA signals show different patterns when using thin liquid barium swallows in comparison to water swallows in the same participants. We hypothesize that HRCA signals will not exhibit significant variations between water and thin liquid barium swallows given the close values of the viscosity properties of both materials. To test this hypothesis, identical-volume water and thin-liquid consistency barium swallows were collected from healthy participants. Tests of statistical significance were performed to check for HRCA signal feature differences between the two types of swallows. Finally, classification models were trained to differentiate between the two types of swallows based on HRCA signal features. If the hypothesis is correct, the classification models will confirm there is no significant variation between the two groups.

## II. Methods and Procedures

### A. Participants and Study Protocol

This study was approved by the institutional review board of the University of Pittsburgh and all participants were provided written informed consent prior to enrollment. Water and barium swallows were performed by 36 healthy community dwelling adults (19 males, 17 females, age 66 ± 8). Participants reported no history of swallowing-related disorders, head and neck surgery, or known neurological diagnoses. Each participant performed 5 water swallows (3 mL by spoon, 1 centipoise viscosity) and 5 swallows (3 mL by spoon) of reconstituted Varibar thin (40% w/v, Bracco Diagnostics Inc., Monroe Twp., NJ) whose viscosity is below 15 centipoise under identical conditions of administration, as a part of a larger study protocol [31], [32]. Trials with water were all completed prior to initiation of videofluoroscopy with barium to avoid the washout (order) effect resulting from using thinner consistency like water after thicker consistency like thin barium. A research SLP administered the boluses to participants for each swallow and the participants were instructed, “Hold this liquid in your mouth and wait until I tell you to swallow.” All swallows were performed in the head neutral position. In total, 185 swallows were included in this analysis (90 barium and 95 water), after excluding swallows with corrupted or unclear VFSS data.

### B. Data Acquisition

For the purposes of this study, we considered swallow trials using two different types of materials: water and thin liquid barium. During water swallows, only HRCA signals were collected. During thin liquid barium swallows, HRCA signals were collected simultaneously with VFSS. A standard fluoroscopy machine (Precision 500D, GE Healthcare, LLC, Waukesha, WI) was used for the thin liquid barium swallows with a pulse rate of 30 PPS. A frame grabber card (AccuStream Express HD, Foresight Imaging, Chelmsford, MA) was used to capture and digitize the video output of the fluoroscopy machine at a rate of 73 FPS. The same HRCA collection equipment was utilized for both types of swallows and used the same hardware configuration described in in prior studies [28], [33]. A contact microphone (model C411L, AKG, Vienna, Austria) and a tri-axial accelerometer (ADXL 327, Analog Devices, Norwood, Massachusetts) were attached to the subjects’ anterior neck. The accelerometer was placed over the cricoid cartilage at midline, a location that has been shown to produce optimal signal quality [34]. The main accelerometer axes were perpendicular to the coronal plane (anterior-posterior), parallel to the cervical spine (superior-inferior), and parallel to the axial-transverse plane (medial-lateral). The microphone was placed lateral to midline from the suprasternal notch towards the right side of the larynx. Resultant signals were hardware bandpass filtered from 0.1 to 3000 Hz [16], [17]. The signals were then digitized at 20kHz utilizing a National Instruments 6210 DAQ.

National Instruments’ LabView was used to synchronize streaming from all sensors and the fluoroscopy machine and to save the streams into a hard drive. Two separate programs were implemented in LabView to record either thin liquid barium swallows with VFSS and fluoro-free water swallows. The first program recorded continuously from the HRCA sensors and the fluoroscopy machine with complete end-to-end synchronization. This guaranteed alignment of swallowing segments for both VFSS videos and HRCA signal recordings for thin liquid barium swallows, using the VFSS images to confirm the onset and offset of each swallow. The second program recorded only output from the HRCA sensors for water swallows with an extra functionality that was used to capture the approximate onset and offset of the swallow. A pushbutton was programmed to create an onset-offset timestamp when pressed and released. This button was pressed/held by a trained researcher, when the command “Swallow,” was given by the administering SLP. The button was subsequently released upon completion of the swallow, denoting approximate swallow offset. The water swallow segments captured at least the entire duration of actual swallows. Information on average duration of swallows for thin liquid barium and water are summarized in table 3.

**TABLE 1:** Summary of Features

Time Domain Features	
Standard deviation	Measure of a signal variance around its mean
Skewness	Measure of the asymmetry of a signal about its mean
Kurtosis	Describes the tailedness/peakness of a signal relative to normal distribution
Information-Theoretic Domain Features	
Lempel-Ziv Complexity	Measure of the randomness of a signal
Normalized Entropy rate	Measure of the degree of regularity of a signal distribution
Frequency Domain Features	
Peak frequency (Hz)	The frequency of maximum power
Spectral centroid (Hz)	The center of mass of the frequency spectrum of a signal
Bandwidth (Hz)	The frequency range of a signal
Time-Frequency Domain Features	
Wavelet entropy	Measure of the disorderly behavior for non-stationary signal

**TABLE 2:** Descriptive Measures Employed in This Study to Assess Classifier Performance

Classifier Performance Measures		Actual Material:		Predictive Values
		Barium	Water	
Predicted Material:	Barium	Correct barium classification (A)	Incorrect barium classification (B)	Barium predictive value $=\frac {A}{A+B}$
	Water	Incorrect water classification (C)	Correct Water classification (D)	Water predictive value $=\frac {D}{C+D}$
Sensitivities		Barium sensitivity $=\frac {A}{A+C}$	Water sensitivity $=\frac {D}{B+D}$	Overall accuracy $=\frac {A+D}{A+B+C+D}$

**TABLE 3:** Relationship Between Quantity, Mean, and Standard Deviation of Duration for Barium and Water Swallows

Descriptive Statistic	Barium	Water
Number of Swallows	90	95
Mean	1.103 seconds	1.468 seconds
Standard Deviation	0.242 seconds	0.375 seconds

### C. VFSS Image Analysis

The onset and offset of thin liquid barium swallows were identified via visual inspection and analysis of the VFSS frames. The onset was defined as the frame during which the bolus head passes the ramus of the mandible. Offset was defined as the frame in which the hyoid bone completes all motion associated with swallowing and returns to resting position [28]. Three expert raters identified the onset and offset of the thin liquid barium swallows in VFSS videos. All expert raters established a priori intra- and inter-rater reliabilities with ICCs over 0.99 using VFSS images that were not included in the dataset under investigation. Additionally, all raters were blinded to participant demographics/history and co-judge ratings to reduce sources of scoring bias.

### D. Signal Preprocessing

All collected signals were downsampled to 4kHz, which retains signal quality while smoothing out any unwanted movement or physiological events that occur during swallowing (e.g. coughing) [16], [35], [36]. The onset and offset of swallows in HRCA signals were calculated based on the onset and offset frames annotated in VFSS videos through using the appropriate sampling conversion. The baseline noise of each sensor, also known as zero-input response of the sensor, was modeled using an auto-regressive model. This model was then used to generate finite impulse response (FIR) filters to remove noise from each part of the HRCA signal (three axes of acceleration and sound signals from the microphone) [36]. All three acceleration signals were individually processed using a fourth order least-squares splines algorithm to reduce low-frequency components produced by participant head movement [37], [38]. Lastly, all signals were denoised using a $10^{th}$ order Meyer wavelet to reduce any remaining noise [39].

### E. Feature Extraction

For a better representation of HRCA signals, nine features were extracted in 4 domains: time, frequency, time-frequency, and information-theoretic. All nine features were extracted from each of the four recorded signals: swallowing sounds from the microphone (MIC), anterior-posterior acceleration (AP), superior-inferior acceleration (SI), and medial-lateral acceleration (ML) to investigate the similarities and differences between the HRCA signals of water and thin liquid barium swallows. These nine features were selected based on prior studies demonstrating their utility for this type of swallowing analysis [18], [33], [40]–[42]. They are summarized in table 1.

### F. Data Analysis

In order to determine whether HRCA signals are different between water and thin liquid barium swallows, linear mixed models have been created for each HRCA signal feature across all 4 signals. The linear mixed models show the statistical significance of each of the features in differentiating between water and thin liquid barium swallows. In other words, the more statistically significant features, the less similar HRCA signals are between water and thin liquid barium swallows. Multiple supervised classifiers were created and tested to determine if HRCA signal features can be used to accurately predict whether a random, unlabeled swallow was of water or thin liquid barium. Three classifiers were tested, including a linear support vector machine (SVM), K-means clustering with two clusters, and a Naïve-Bayes classifier. The analyzed data consisted of 36 total features and 9 unique features from the 4 separate signals. Each classifier employed principal component analysis (PCA) with 8 principal components. Dimensionality reduction to eight principal components consistently explained greater than 97% of the variability of the input data.

The total number of swallows available for analysis was limited, so a validation strategy was applied. Holdout validation is employed using a train-test split of 70%-30%. The holdout validation strategy is repeated 2,000 times by random selection of through randomly choosing the train and test splits across the data each time for each iteration. The training and testing data is fully randomized for 2,000 trials. The classification accuracies are averaged over all trials to obtain true accuracy measures.

In medical diagnostic applications, sensitivity (true positives over true positives and false negatives) and specificity (true negatives over true negatives and false positives) are routinely utilized. This study consists of two distinct output classes, rather than a direct positive and negative outcome. This study introduces distinct accuracy measures which are used in analysis. Overall accuracy (accurate classifications over all swallows), sensitivity to barium (accurate barium classifications over all barium swallows), sensitivity to water (accurate water classifications over all water swallows), barium predictive value (accurate barium classifications over all barium classifications), and water predictive value (accurate water classifications over all water classifications). The performance measures employed in this study are illustrated in table 2. All statistical analysis was performed in SAS (SAS Institute, Inc., Cary, North Carolina) and all supervised classifiers were implemented and tested in MATLAB (The MathWorks, Inc., Natick, Massachusetts).

## III. Results

This analysis included feature data only from those participants who completed both water and thin liquid barium swallows as part of the study protocol. The analyzed data consisted of 185 total swallows, with 90 barium swallows and 95 water swallows from a total of 19 participants. HRCA signals collected for water swallows were significantly longer than barium swallows, as noted in Table 3. Figure 2 illustrates each axis of the raw HRCA signals for a single participant.

**FIGURE 1.**
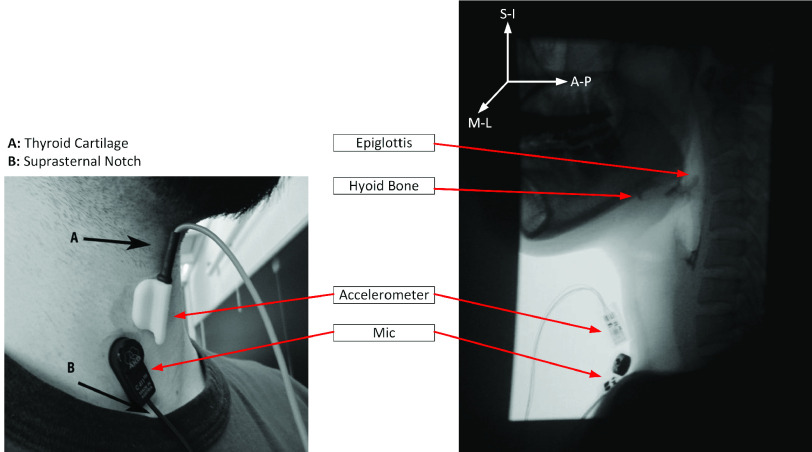
Placement of the tri-axial accelerometer and contact microphone.

**FIGURE 2.**
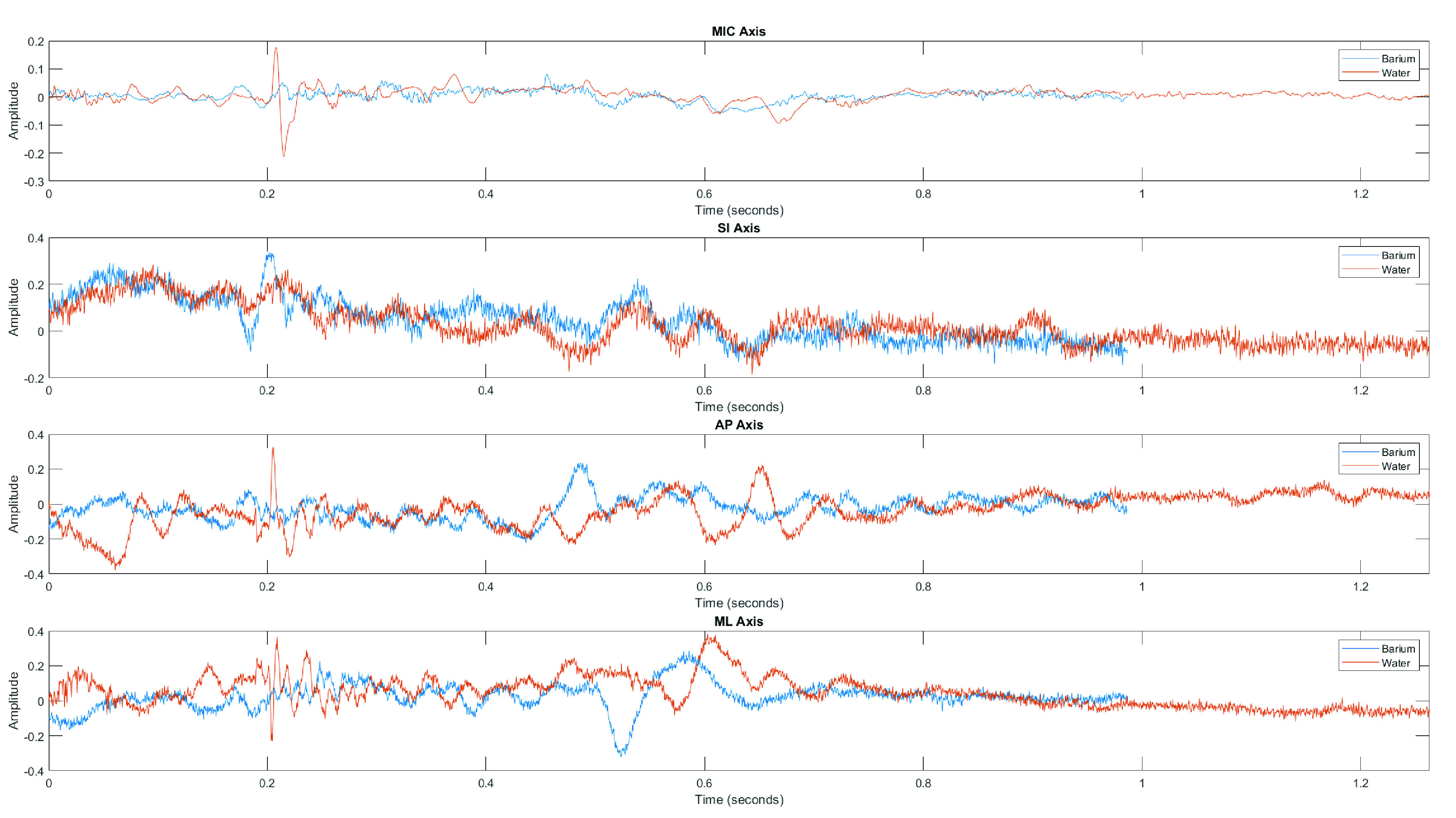
Comparison of swallowing sounds and SI, AP, and ML acceleration between a barium swallow and water swallow for a single participant (Blue = Barium $||$ Orange = Water).

The HRCA signals were summarized at the participant level. The descriptive statistics for all HRCA signal features (mean ± standard deviation), are depicted in Table 4.

**TABLE 4:** Descriptive Statistics (Mean and Standard Deviation) of All HRCA Features for MIC, AP, SS, and ML Axes for Both Barium and Water Swallows

Extracted Features	MIC		AP		SI		ML	
Time Domain	Barium	Water	Barium	Water	Barium	Water	Barium	Water
Standard Deviation	0.011 ± 0.014	0.012 ± 0.010	0.010 ± 0.016	0.011 ± 0.015	0.022 ± 0.028	0.024 ± 0.025	0.010 ± 0.011	0.012 ± 0.012
Skewness	$-0.543\,\,\pm \,\,0.743$	$-0.825\,\,\pm \,\,1.124$	$-0.185\,\,\pm \,\,1.167$	$-0.002\,\,\pm \,\,0.509$	$-0.140\,\,\pm \,\,0.776$	$-0.029\,\,\pm \,\,0.47$	0.301 ± 0.831	0.315 ± 0.648
Kurtosis	18.646 ± 17.230	28.804 ± 21.839	13.989 ± 36.581	6.024 ± 13.319	8.274 ± 8.543	8.046 ± 7.883	10.332 ± 16.147	7.936 ± 9.468
Information-Theoretic Domain								
Lempel-Ziv Complexity	0.251 ± 0.057	0.199 ± 0.055	0.224 ± 0.214	0.213 ± 0.199	0.152 ± 0.053	0.145 ± 0.059	0.114 ± 0.031	0.107 ± 0.026
Normalized Entropy Rate	0.909 ± 0.038	0.936 ± 0.022	0.862 ± 0.199	0.891 ± 0.148	0.956 ± 0.010	0.964 ± 0.011	0.961 ± 0.009	0.966 ± 0.009
Frequency Domain								
Peak Frequency (Hz)	9.541 ± 10.085	7.345 ± 8.221	12.741 ± 28.258	0.966 ± 0.409	4.875 ± 4.398	5.118 ± 4.923	2.206 ± 2.671	1.885 ± 1.059
Spectral Centroid (Hz)	63.399 ± 28.587	62.152 ± 28.025	45.395 ± 61.979	25.257 ± 31.512	15.701 ± 18.241	12.667 ± 11.05	35.708 ± 78.885	24.891 ± 39.199
Bandwidth (Hz)	87.298 ± 35.314	86.102 ± 35.349	78.578 ± 69.379	57.405 ± 51.251	33.635 ± 30.592	23.339 ± 15.294	78.019 ± 113.267	57.977 ± 80.418
Time-Frequency Domain								
Wavelet Entropy	1.300 ± 0.478	1.720 ± 0.488	0.211 ± 0.153	0.238 ± 0.16	0.509 ± 0.410	0.842 ± 0.59	0.420 ± 0.459	0.610 ± 0.567

The null hypothesis proposed in this study states that there is no statistically significant difference between the HRCA signals of a water swallow and a barium swallow. A rejection of the null hypothesis proposes there is a significant difference in corresponding HRCA signal features for barium and water swallows. The linear mixed models use a confidence level of 0.95 ( $\alpha =0.05$). A p-value less than the significance level of 0.05 indicates a clear rejection of the null hypothesis for the corresponding HRCA signal feature. The linear mixed models demonstrated that 28 HRCA signal features exhibited no systematic bias or difference between water and barium swallows. However, 8 HRCA signal features exhibited statistically significant differences between water and barium swallows. Table 5 depicts only the HRCA signal features that exhibit statistically significant differences between water and barium swallows. Table 6 depicts the relationship between HRCA axis and the number of statistically significant features, while table 7 depicts the relationship between the domain of HRCA signals and the number of statistically significant features.

**TABLE 5:** Depiction of Rejected HRCA Features and Corresponding P-Value

Rejected Feature	P-Value
MIC Lempel-Ziv Complexity	0.0005
MIC Normalized Entropy Rate	0.0043
MIC Wavelet Entropy	0.0060
MIC Kurtosis	0.0089
SI Wavelet Entropy	0.0146
SI Normalized Entropy Rate	0.0291
ML Standard Deviation	0.0387
AP Normalized Entropy Rate	0.0395

**TABLE 6:** Relationship Between Axis of HRCA Signal and the Number of Rejected Features

Axis of Rejected Feature	Number of Features
MIC	4
AP	1
SI	2
ML	1

**TABLE 7:** Relationship Between Domain of HRCA Signal and the Number of Rejected Features

Domain of Rejected Feature	Number of Features
Time	2
Information-Theoretic	4
Frequency	0
Time-Frequency	2

With or without dimensionality reduction using PCA, none of the classifiers demonstrated high overall accuracy, sensitivity, or predictive values. Low accuracy demonstrates the classifiers cannot properly differentiate the HRCA signal features between a water swallow and a barium swallow. Dimensionality reduction had no effect on the K-means classifier, marginal effect on the Naïve Bayes classifier, and greatly reduced all performance measures, with the exception of water sensitivity, for the SVM classifier.

Without PCA, the SVM classifier had the highest overall accuracy, while exhibiting similar measures for barium and water sensitivity and predictive value. The Naïve-Bayes and K-means classifiers made correct predictions approximately 50% of the time. The barium sensitivity for these two classifiers is significantly lower than the water sensitivity. The predictive value for barium and predictive value for water are nearly equal with performance similar to the overall accuracy.

Using PCA for dimensionality reduction, all three classifiers performed similarly. Naïve-Bayes and K-means performed similarly with and without PCA. Each classifier was only able to make a correct prediction around half the time. The barium sensitivity for all three classifiers is significantly lower than the water sensitivity. The predictive value for barium and predictive value for water are nearly equal with performance similar to the overall accuracy.

Table 8 and Table 9 illustrate all five performance measures for all three classifiers without dimensionality reduction and with dimensionality reduction using principal component analysis, respectively.

**TABLE 8:** Performance Measures of Classifiers to Detect Swallow Material Using HRCA Signal Features of Barium and Water Swallows Without Dimensionality Reduction

Classifier	Overall Accuracy	Barium Sensitivity	Water Sensitivity	Barium Predictive Value	Water Predictive Value
SVM	63.186%	61.263%	65.041%	63.125%	63.813%
Naïve Bayes	58.853%	29.519%	87.139%	69.924%	56.267%
K-Means	50.980%	31.252%	70.004%	57.515%	48.989%

**TABLE 9:** Performance Measures of Classifiers to Detect Swallow Material Using HRCA Signal Features of Barium and Water Swallows With Dimensionality Reduction Using PCA

Classifier	Overall Accuracy	Barium Sensitivity	Water Sensitivity	Barium Predictive Value	Water Predictive Value
SVM	52.415%	28.565%	75.414%	54.765%	52.250%
Naïve Bayes	53.371%	24.109%	81.588%	57.284%	52.686%
K-Means	51.078%	30.798%	70.634%	57.444%	49.198%

## IV. Discussion

The results indicate that there are no significant differences between 28 of 36 the HRCA signal features of swallows using 3mL water or 3mL of thin barium liquid, indicating that signals obtained during bedside screening with water would predict signal features performance that would be collected during a VFSS study. This finding supports the efficacy of HRCA as an accurate OPD screening tool.

This study investigated the differences in HRCA signals between thin liquid barium swallows and water swallows by utilizing linear mixed models created for all 36 HRCA signal features. Each linear mixed model operated with the null hypothesis that there is no difference between HRCA signals of a thin liquid barium swallow and a water swallow. The results showed that the null hypothesis is rejected for 8 of 36 features and not rejected for 28 of 36 features. The 8 features with rejected null hypothesis are depicted in Table 5, with the frequency of rejected features for each axis and domain represented in Table 6 and Table 7, respectively. Four of these features emanate from swallowing sounds alone. All the swallows analyzed in this study derive from healthy participants. As healthy swallows ordinarily do not involve aspiration or produce noise, such as choking or coughing, it is expected that amplitude of swallowing sounds will be minimal. Conceivably, differences in swallowing sounds between thin liquid barium swallows and water swallows from a small sample size may be expected. A larger sample size may minimize this finding.

As for the domain, significant findings were present in half (2) of the information-theoretic features and half (4) of the time-frequency features. These six features account for three quarters of all HRCA features with significant results. Table 4 shows that the Lempel-Ziv complexity is lower, the normalized entropy rate is higher, and the wavelet entropy is higher for all 4 axes (MIC,AP,SI,ML) of water swallows compared with barium. Lempel-Ziv complexity is a measure of the predictability of a signal [36], [40]. A larger value of the normalized entropy rate feature, as employed in this study, demonstrates more regularity in the signal. Wavelet entropy indicates the degree of order in a signal [43]. A higher wavelet entropy demonstrates more disordered signal. The thin liquid barium swallows were segmented by expert raters using frame-by-frame VFSS. This method of precise segmentation ensures that the signal duration of a barium swallow contains information from onset to offset of swallow only. Conversely, water swallows were segmented based on verbal cue and visual observation of swallowing behavior of the participant. Using a pushbutton integrated in the collection program, a trained operator determined the onset of water swallows when the command of “swallow” is verbally initiated by the researcher and the offset when the participant is deemed to complete the swallow by visual observation. Considering the differences in swallow duration for thin liquid barium versus water swallows, this is a likely explanation for this feature difference. The HRCA signals from the analyzed water swallows are significantly longer. Given that the viscosity of water is comparable to the viscosity of Varibar thin and the trials are conducted in the same systematic, protocol-driven, controlled environment, true swallow duration (from onset to offset) for each material is presumably equivalent. Thus, there are more non-swallow signals present in the segments water swallows. This extra time will add an increased number of small movements and sounds, consequently affecting these features.

Whether or not PCA for dimensionality reduction was employed, the overall accuracy of the classifiers was nearly 50%. With the null hypothesis stating that there is no difference between barium and water swallows, this accuracy metric provides support to accept the null hypothesis. However, barium sensitivity was generally low, and water sensitivity was high. As defined in table 2, a barium swallow was correctly classified infrequently while a water swallow was correctly classified frequently.

To better distinguish the predictability of barium and water swallows, predictive value is an appropriate, alternative characteristic. Predictive value is the ratio of correct classifications for a given material to all classifications for the given material. For example, barium predictive value estimates the likelihood that the classified swallow was truly completed with barium when a barium swallow was predicted. Barium and water predictive values for the 3 classifiers, with and without dimensionality reduction, are nearly equal, approximately 50%. These performance measures, along with the overall accuracy of the classifiers at approximately 50%, demonstrate an unlabeled set of HRCA signals will essentially be predicted randomly as either performed with barium or water. This metric demonstrates there is no significant difference between the HRCA signals of a barium swallow and a water swallow.

## V. Conclusion

This study investigated whether there are statistically significant differences in HRCA signal features between water and thin liquid barium swallows. The purpose of this analysis was part of a preliminary determination as to the feasibility and clinical relevance of using an HRCA based system as a potential method for enhanced bedside swallowing screening and/or an adjunct to clinical swallowing assessment, extending access of screening and diagnostic OPD capabilities to underserved patients. These results indicate no significant difference between HRCA signals of barium and water swallows. Of note, though there is no systematic difference between the HRCA signals of a barium swallow and water swallow, there is insufficient evidence to confirm similarity between the HRCA signals. HRCA signals between both materials do not exhibit differences significant enough to rule out similarity; however, the analyzed data cannot confirm the hypothesis that there is no difference between the HRCA signals of a thin liquid barium swallow and a water swallow. Replication with a larger data set is necessary to sort these remaining questions.

The dataset utilized for analysis and classification consisted of fewer than 100 swallows per material and fewer than 20 participants. Even with utilization of holdout validation for more accurate classification, it is not feasible to conclusively state that these materials are similar, with respect to HRCA signals. analyzed dataset cannot conclusively confirm similarity. To further test the hypothesis and determine which HRCA signal features exhibit similarity or difference, more data must be collected and analyzed to determine whether there is a clear correlation between the HRCA signals of a barium swallow and water swallow.
